# Pharmacology and pharmacogenetics of prednisone and prednisolone in patients with nephrotic syndrome

**DOI:** 10.1007/s00467-018-3929-z

**Published:** 2018-03-16

**Authors:** Anne M. Schijvens, Rob ter Heine, Saskia N. de Wildt, Michiel F. Schreuder

**Affiliations:** 1 Department of Pediatric Nephrology, Radboud University Medical Center, Radboud Institute for Molecular Life Sciences, Amalia Children’s Hospital, 804, P.O. Box 9101, 6500 HB Nijmegen, The Netherlands; 2 Department of Pharmacy, Radboud University Medical Center, Radboud Institute for Health Sciences, Nijmegen, The Netherlands; 3 Department of Pharmacology and Toxicology, Radboud University Medical Center, Nijmegen, The Netherlands; 4 Intensive Care and Department of Pediatric Surgery, Erasmus MC-Sophia Children’s Hospital, Rotterdam, The Netherlands

**Keywords:** Nephrotic syndrome, Pharmacogenetics, Prednisolone, Prednisone, Glucocorticoids, Pharmacology

## Abstract

Nephrotic syndrome is one of the most common glomerular disorders in childhood. Glucocorticoids have been the cornerstone of the treatment of childhood nephrotic syndrome for several decades, as the majority of children achieves complete remission after prednisone or prednisolone treatment. Currently, treatment guidelines for the first manifestation and relapse of nephrotic syndrome are mostly standardized, while large inter-individual variation is present in the clinical course of disease and side effects of glucocorticoid treatment. This review describes the mechanisms of glucocorticoid action and clinical pharmacokinetics and pharmacodynamics of prednisone and prednisolone in nephrotic syndrome patients. However, these mechanisms do not account for the large inter-individual variability in the response to glucocorticoid treatment. Previous research has shown that genetic factors can have a major influence on the pharmacokinetic and dynamic profile of the individual patient. Therefore, pharmacogenetics may have a promising role in personalized medicine for patients with nephrotic syndrome. Currently, little is known about the impact of genetic polymorphisms on glucocorticoid response and steroid-related toxicities in children with nephrotic syndrome. Although the evidence is limited, the data summarized in this study do suggest a role for pharmacogenetics to improve individualization of glucocorticoid therapy. Therefore, studies in larger cohorts with nephrotic syndrome patients are necessary to draw final conclusions about the influence of genetic polymorphisms on the glucocorticoid response and steroid-related toxicities to ultimately implement pharmacogenetics in clinical practice.

## Introduction

Nephrotic syndrome is one of the most common glomerular disorders in children and affects 1–7 per 100,000 children per year (Dutch data 1.52/100,000) with a male predominance (2:1) [1]. The disease is characterized by the triad of severe proteinuria, hypoalbuminemia, and edema. Glucocorticoids have been the cornerstone of the treatment of childhood nephrotic syndrome for over 60 years, as over 80–90% of the patients achieve complete remission with prednisone or prednisolone treatment [2]. Unfortunately, 80% of these patients will have one or several relapses and will need additional courses of glucocorticoid therapy. Furthermore, approximately 10% of children with nephrotic syndrome are steroid resistant and do not respond to the standard steroid treatment regimen. Treatment guidelines for the first manifestation and a relapse of steroid-sensitive nephrotic syndrome are mostly standardized and based on practice guidelines rather than clinical trials [3]. As the optimal glucocorticoid dosing regimens for childhood nephrotic syndrome are still under debate and large-scale clinical trials are lacking, current clinical practice among physicians is variable [4], especially in the treatment of subsequent relapses and the choice of second-line immunosuppressive drugs. Unfortunately, the variability in the treatment of nephrotic syndrome is mostly based on the protocol preference of the physician, rather than the individual characteristics of the patient. Therefore, clinical trials are needed to develop international treatment guidelines with recommendations for several aspects of the treatment of nephrotic syndrome. Recently, a nationwide study in the Netherlands showed that duration of corticosteroids for the initial presentation had no impact on subsequent relapses [5]. Furthermore, a recently published abstract of the PREDNOS study indicated no clinical benefit associated with an extended steroid course for the initial presentation in UK children [6]. Currently, a few clinical trials are underway to further investigate the optimal dosing regimens for both the first manifestation (https://clinicaltrials.gov/ct2/show/NCT02649413?recrs=abdf&cond=Nephrotic+Syndrome&age=0&draw=2&rank=13) as well as relapses of nephrotic syndrome [7].

Large inter-individual variation is present in children with nephrotic syndrome regarding both the clinical course of disease and the intensity and spectrum of side effects of its treatment. Nephrotic syndrome is characterized by podocyte foot process effacement; however, the exact mechanism of disease is still largely unknown and often debated. Several etiologies have been investigated over the years, and different subgroups of the disease are likely to have a different pathogenesis [8, 9]. Damage to the filtration barrier can be caused by genetic defects primarily affecting podocytes. Patients with an underlying genetic defect are often primary steroid resistant, and to date, 53 genes associated with steroid-resistant nephrotic syndrome have been identified [10]. Furthermore, a substantial proportion of the patients is likely to have an immune-mediated circulating factor disease. These patients are also often steroid resistant, but screen negative for the known steroid-resistant nephrotic syndrome genes. The existence of a circulating permeability factor would explain the rapid recurrence of proteinuria after kidney transplantation in some patients with nephrotic syndrome [11, 12]. Many candidates have been identified over the years; however, the definitive factor remains to be discovered [13]. Lastly, involvement of the immune system in the pathogenesis of nephrotic syndrome is highly suspected as relapses often occur after the immune system is triggered by an infection, allergy, or vaccination, and glucocorticoid treatment is effective in most patients. Nephrotic syndrome has been considered to be a T cell disorder based on several observations, including remission following measles infection, the association with Hodgkin disease, and the response to immunosuppressive drugs [9]. Furthermore, in the last few years, a potential role for B cells has been proposed as well due to the effectiveness of B cell depletion with rituximab in patients with nephrotic syndrome [9, 14].

All in all, most children with nephrotic syndrome have a minimal change disease [15] and, therefore, the large inter-patient variability cannot fully be attributed to the disease histology. Previous research has indicated that pharmacogenetics can have an influence on both pharmacokinetics (PK) and pharmacodynamics (PD) of the individual patient [16]. Genetic factors influencing the individual pharmacokinetic and pharmacodynamic profile may account for 20–95% of the variability in the efficacy and side effects of medication [17]. Each of the processes involved in PK and PD can potentially be influenced by a clinical significant genetic variation [18]. Therefore, pharmacogenetics may have a promising role in personalized medicine. By implementing pharmacogenetics in the clinical work-up of the patients, this may ultimately lead to individualized drug therapy to maximize drug efficacy and minimize drug toxicity. In the era of precision medicine, however, current knowledge on the influence of pharmacogenetics on the steroid response in nephrotic syndrome is limited [19].

This review describes the mechanisms of glucocorticoid action and clinical PK and PD of prednisone and prednisolone in nephrotic syndrome patients. Furthermore, the current data available on pharmacogenetics of prednisone and prednisolone in patients with nephrotic syndrome is summarized and areas for future research to improve individualization of glucocorticoid therapy in children with nephrotic syndrome are identified.

## Mechanisms of glucocorticoid action

Glucocorticoids are potent anti-inflammatory and immunosuppressant drugs. The effects of glucocorticoids are mediated by both genomic and non-genomic mechanisms. Genomic mechanisms implicate the activation or repression of specific genes encoding anti- and pro-inflammatory proteins. As a consequence of the time-consuming mRNA transcription and translation, the genomic glucocorticoid action is characterized by a slow onset of the response. In contrast, non-genomic mechanisms do not influence gene expression and have a rapid onset and a short duration of the effect [20].

### Genomic mechanisms (Fig. 1)

Glucocorticoids are, like other steroid hormones, lipophilic molecules that can easily diffuse across the cell membrane and bind to the glucocorticoid receptors (GRs) in the cytoplasm [21]. The inactive GR is bound to a chaperone protein complex to keep the inactive GR in the correct folding for hormone binding and prevent nuclear localization of unoccupied GRs [19, 22, 23]. When glucocorticoids enter the cell after passive diffusion and bind to the GR in the cytoplasm, a glucocorticoid receptor/glucocorticoid (GR/GC) complex is formed. Subsequently, the chaperone protein complex dissociates, allowing the transfer of the GR/GC complex into the nucleus. The mechanism of nuclear translocation involves the nuclear import proteins importin-α and importin-13 (IPO13) [24]. After entering the nucleus, the activated GR/GC complex binds to the DNA or interacts with co-activator complexes. The activated GR/GC complex exerts its anti-inflammatory and immunosuppressive effects by increased expression of anti-inflammatory genes (transactivation) and decreased expression of pro-inflammatory genes (transrepression) [23, 25]. Furthermore, the GR/GC complex can, either directly or indirectly, interact with pro-inflammatory transcription factors nuclear factor κB (NF-κB) and activator protein 1 (AP-1) and thus reduce their activity [20, 26].

**Fig. 1:**
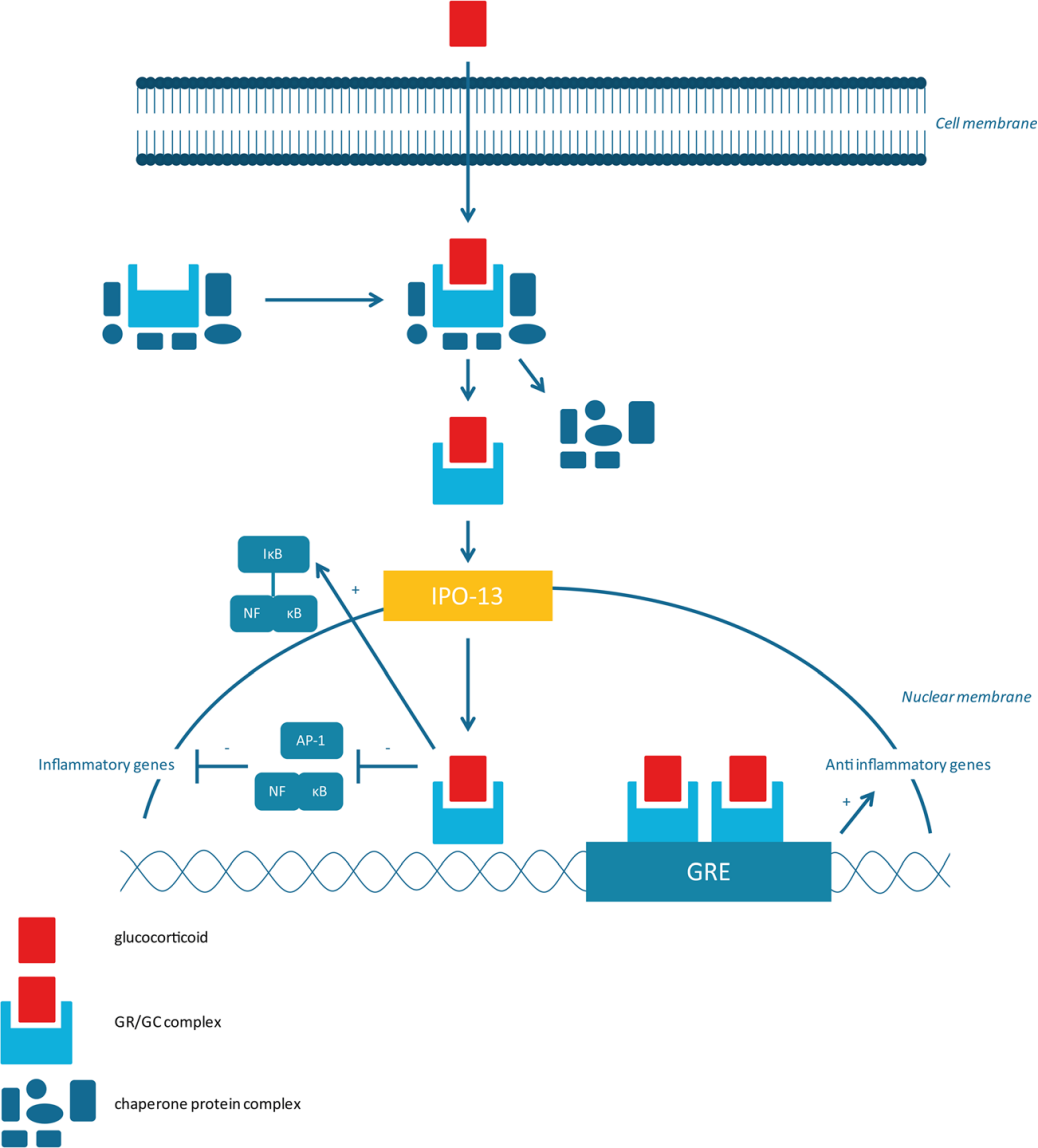
Molecular mechanisms of glucocorticoid action. AP-1, activator protein 1; IκB, inhibitor of kappa B; IPO-13, importin-13; NF-κB, nuclear factor κB; GRE, glucocorticoid response elements

### Non-genomic effects

The non-genomic mechanisms of glucocorticoid action remain largely undefined. Glucocorticoids affect the physicochemical property of cell membranes, directly or through binding to intracellular or membrane-bound GRs [27]. The effects result in the inhibition of inflammatory cell function [28]. Another hypothesis is that non-genomic effects are mediated after GR/GC binding. When glucocorticoids bind to the GR, the aforementioned chaperone proteins are released. The release of signaling molecules from the multiprotein complex is also considered to be responsible for rapid glucocorticoid effects [29].

## Pharmacokinetics

Pharmacokinetics describes the study of what the body does to a drug. PK involves the processes of absorption, distribution, metabolism, and excretion, often abbreviated as ADME.

### Prednisone and prednisolone

For the treatment of nephrotic syndrome, both prednisone and prednisolone are frequently used glucocorticoids. Prednisone is a prodrug of prednisolone and is bioactivated by the enzyme 11β-hydroxysteroid dehydrogenase (11β-HSD)-1. The conversion of prednisone into prednisolone occurs rapidly, and plasma concentrations of both substances reach their peaks at approximately 0.5–3 h after prednisone administration, in both patients with and without nephrotic syndrome [20, 30–33]. In addition, inter-conversion is present between both substances and this varies with time and dose. However, prednisolone concentrations are four- to tenfold higher than prednisone concentrations. In children with nephrotic syndrome, both in the active phase and in remission, similar ratios were found, which indicates that nephrotic syndrome does not influence the conversion from prednisone into prednisolone [30–32, 34].

### Absorption

Both prednisone and prednisolone are well absorbed after oral administration. A variable systemic bioavailability for prednisone and prednisolone has been reported in the literature: 84 ± 13 and 99 ± 8%, respectively [20]. The high variability in bioavailability is most likely mainly based on inter-individual differences rather than the choice of prednisone or prednisolone [35]. In the Kidney Disease: Improving Global Outcomes (KDIGO) guideline for glomerulonephritis, prednisone and prednisolone are considered equivalent and identical dosages are used for the standardized treatment regimens for patients with nephrotic syndrome [3]. Peak plasma concentrations are reached at approximately 0.5–3 h after administration. Food intake is generally considered to prolong the time to maximum drug concentration (*T*_max_), but not the extent of drug absorption [36].

#### Nephrotic syndrome

**Fig. 2:**
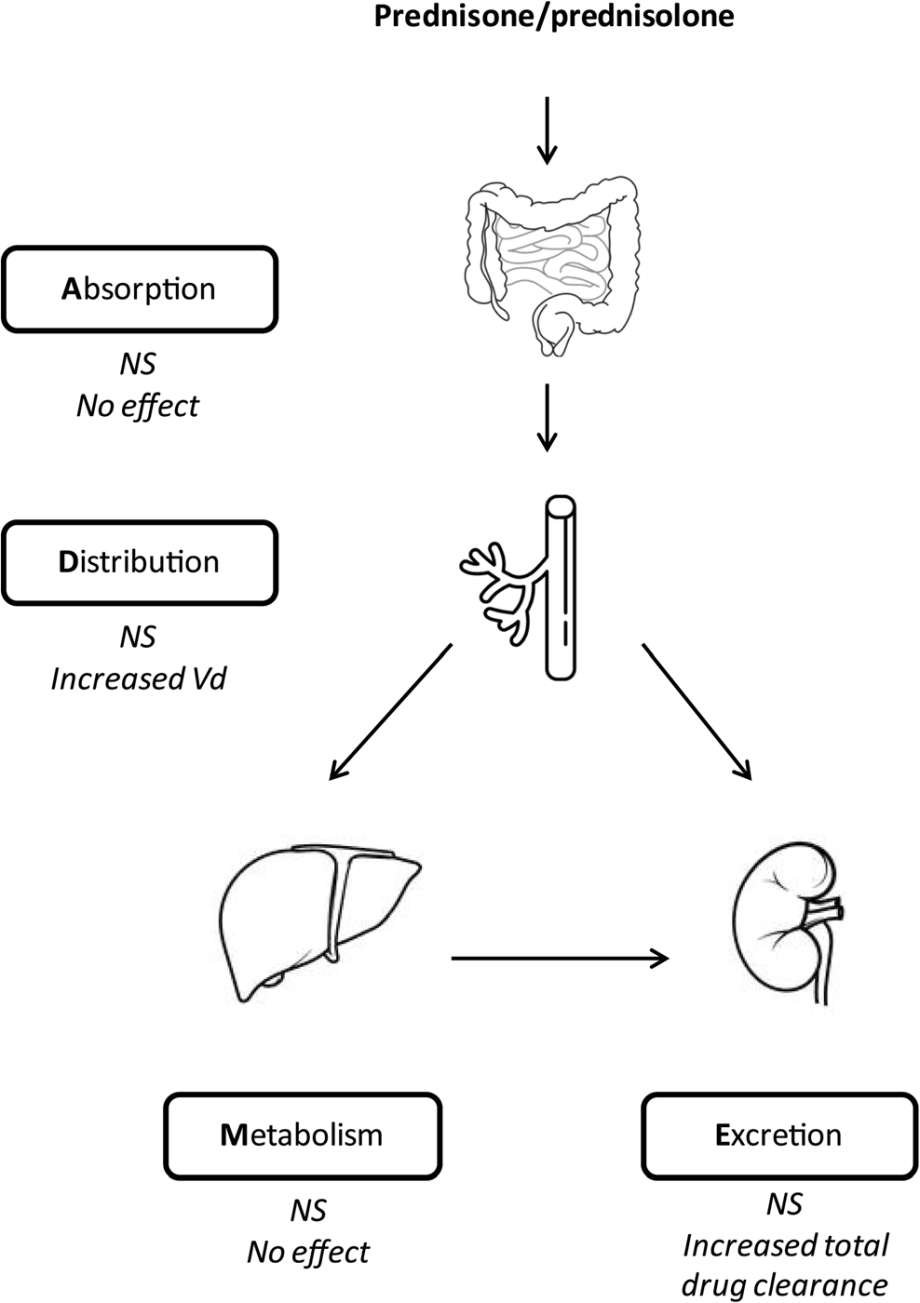
ADME prednisone/prednisolone in patients with nephrotic syndrome

In patients with nephrotic syndrome, a similar bioavailability profile has been described, indicating that the nephrotic state does not influence the absorption of prednisolone and prednisone [34, 37] (Fig. 2).

### Distribution

The volume of distribution of prednisolone and prednisone in adults is 0.64 l/kg [38] and 0.4–1.0 l/kg [39], respectively. The total plasma PK of prednisolone and prednisone appears non-linear, due to non-linear protein binding. The protein-free PK, however, is linear. Non-linear protein binding is most evident in the dose range between 5 and 50 mg [32]. Prednisolone binds to the glycoprotein transcortin (i.e., corticosteroid-binding globulin) and to albumin. Transcortin is a small (50–60 kDa), high-affinity, low-capacity protein with normal blood concentrations of 32.0–50.0 mg/l. In contrast, albumin (60 kDa) has a low affinity but high capacity [32, 40]. Protein binding of prednisolone decreases non-linearly from approximately 95% at plasma concentrations of 200 μg/l down to 60–70% at plasma concentrations of 800 μg/l. Subsequently, a dose-dependent increase in the volume of distribution and drug clearance is observed [20].

#### Nephrotic syndrome

**Fig. 3:**
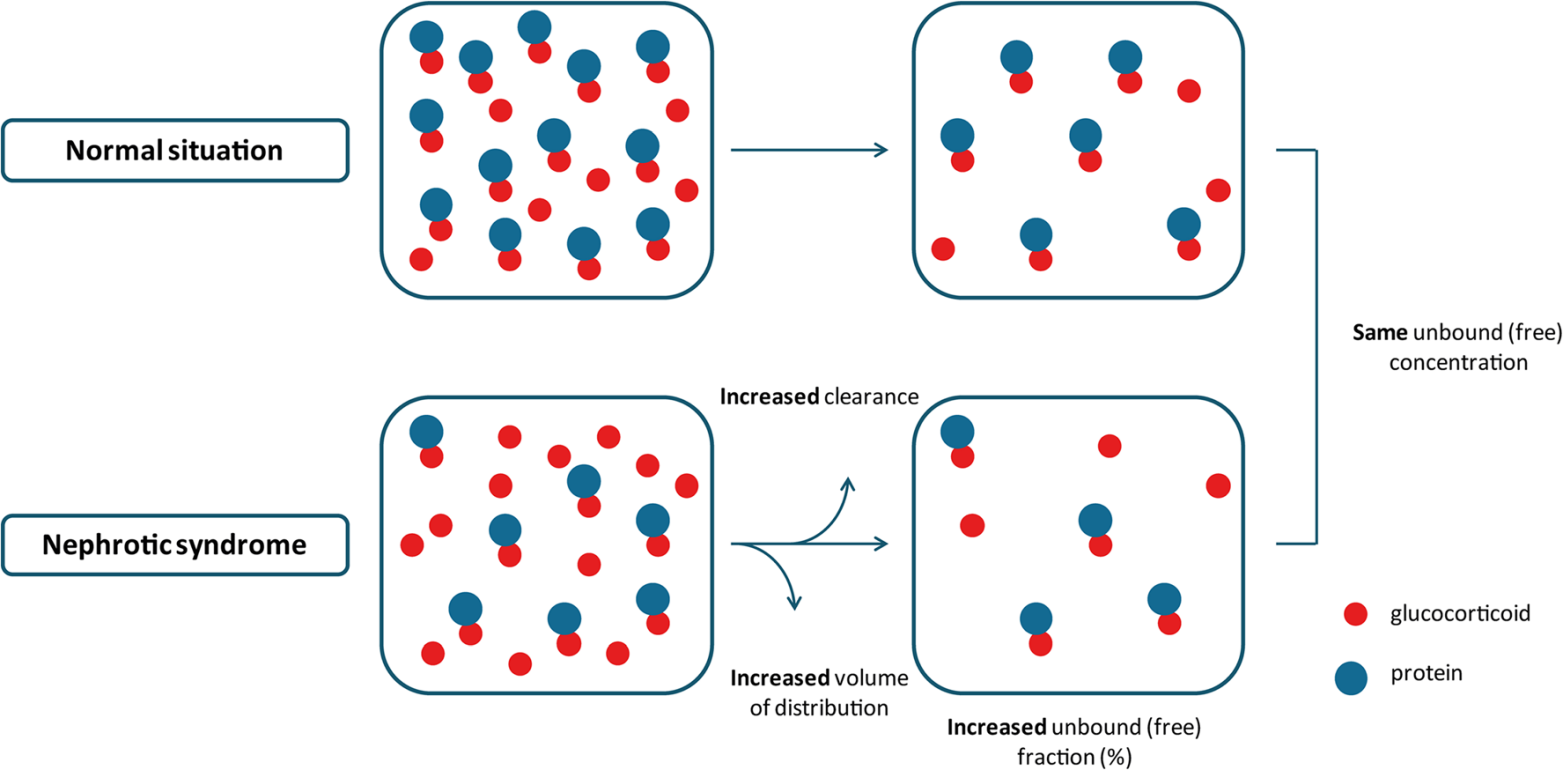
Unchanged unbound (free) concentration in patients with nephrotic syndrome

Patients with nephrotic syndrome have decreased serum albumin and transcortin levels in the active phase of disease, leading to a decreased protein binding of prednisone and prednisolone [37, 38]. When the unbound fraction increases due to less protein binding, the drug is eliminated more rapidly and the volume of distribution of total prednisolone increases as the displaced drug spreads out. The end result is an initial increase in unbound concentration, a decrease in total drug concentration, and no change in the steady-state unbound concentration (Fig. 3). These findings underline the necessity of evaluating unbound concentrations in pharmacological research. Recently, Teeninga et al. showed a good correlation between salivary prednisolone levels and free serum prednisolone levels in healthy volunteers and pediatric nephrotic syndrome patients, indicating the potential use of saliva as a non-invasive and feasible method for drug monitoring of prednisolone [41].

Several pharmacokinetic studies performed in both pediatric [30, 31, 34, 40] and adult [37, 38, 42] nephrotic syndrome patients have confirmed the increase in unbound fraction, but unchanged steady-state unbound concentration of prednisolone. Moreover, pharmacokinetic studies performed in children with nephrotic syndrome both in the active phase of disease and in remission showed that free prednisolone concentrations during the active phase did not significantly differ from those observed during remission [31]. Furthermore, pharmacokinetic studies for other highly protein-bound drugs showed similar results with an increase of total volume of distribution, total clearance, and free fraction of the drugs, but unchanged free drug concentrations in steady state [43].

### Metabolism

Intracellular metabolism by 11β-HSD controls the availability of prednisolone for binding to the glucocorticoid and mineralocorticoid receptors. Two types of 11β-HSD are present in the body. 11β-HSD-1 acts primarily as a reductase and converts the inactive prednisone into the active prednisolone. 11β-HSD-2 acts primarily as an oxidase and converts prednisolone to prednisone, thereby protecting the mineralocorticoid receptor from occupation by cortisol and prednisolone [44]. The undesired mineralocorticoid effects of glucocorticoid treatment will most likely be pronounced when the capacity of 11β-HSD-2 is exceeded. Therefore, the mineralocorticoid effects of glucocorticoids might depend on the administration scheme. A low glucocorticoid dose leading to concentrations just above the protective capacity of 11β-HSD-2 is expected to have reduced mineralocorticoid effects when administered as two dose fractions, because both concentration peaks would not exceed 11β-HSD-2 capacity. In contrast, higher glucocorticoid doses, exceeding the 11β-HSD-2 capacity and even leading to saturation of the mineralocorticoid receptor, are expected to have enhanced mineralocorticoid effects when administered as two dose fractions, because the total time during which mineralocorticoid receptors are occupied would be prolonged.

Prednisolone and prednisone are primarily cleared from the body by hepatic metabolism involving phase I and phase II reactions. The most important enzyme system of phase I metabolism is cytochrome P450. However, for the prednisone/prednisolone metabolism, the degree of involvement of specific cytochrome P450 (CYP)3A isoenzymes has not been fully elucidated yet. Nevertheless, co-administration of the strong CYP3A4 inhibitor ketoconazole is shown to increase total and unbound prednisolone concentrations in plasma by about 50% due to a decreased clearance [45]. In line with this, previous research has shown co-administration of enzyme inducers to cause an increased clearance and decreased half-life of prednisolone [46–48].

Furthermore, in vitro data suggest that prednisolone is also a substrate of P-glycoprotein. P-glycoprotein is an ATP-dependent efflux membrane transporter, which is widely distributed throughout the body and highly expressed in the small intestine and kidneys. Expression of P-glycoprotein in the intestinal epithelium limits the absorption of drug substrates from the gastrointestinal tract. Therefore, theoretically, co-administration of P-glycoprotein inhibitors could increase glucocorticoid absorption and oral bioavailability and might affect glucocorticoid distribution [20]. A previous study conducted in adult renal patients, however, showed a normal metabolism of prednisolone in patients treated with cyclosporine, which is a P-glycoprotein inhibitor [49].

#### Nephrotic syndrome

For patients with nephrotic syndrome, different dosing regimens have been investigated. Single daily dosing appears to be as effective as multiple daily dosing in maintaining remission in children [50]. Serious side effects, including hypertension, Cushingoid appearance, and obesity, were less common in patients receiving the single daily dose compared to patients receiving divided doses [50]. We hypothesize that this might be due to continuously exceeding the 11β-HSD-2 capacity in case of multiple daily dosing as a consequence of the high doses of steroids given in patients with nephrotic syndrome.

Whether P-glycoprotein inhibitors are able to increase glucocorticoid availability in patients with nephrotic syndrome is unknown. In addition, glucocorticoids are known to affect the PK of other drugs by enzyme induction as well by inducing CYP3A4 [51] and P-glycoprotein [52]; however, the clinical importance of enzyme induction by prednisone/prednisolone is largely unknown [20].

### Excretion

P-glycoprotein is also located in the liver and kidney, resulting in enhanced excretion of drug substrates into bile and urine, respectively. In this case, co-administration of P-glycoprotein inhibitors could potentially result in decreased excretion of prednisone/prednisolone and an increased retention time [53]. The previously mentioned pharmacokinetic study in renal transplant patients, however, also showed a normal metabolic and renal clearance of prednisolone in the presence of cyclosporine [49]. Elimination half-lives (*T*_1/2_) in adults are 3.3 ± 1.3 h for prednisone and 3.2 ± 1.0 h for prednisolone [20]. In a pharmacokinetic study performed in children, lower mean elimination half-lives of 2.2 ± 0.5 h were found [33].

#### Nephrotic syndrome

In the aforementioned study, children with a variety of diseases (e.g., nephrotic syndrome, asthma, systemic lupus erythematosus, congenital virilizing adrenal hyperplasia) were included. Children with congenital virilizing adrenal hyperplasia were considered to be comparable to normal subjects. No difference regarding elimination half-lives was found between this group and the children with nephrotic syndrome [33]. Similarly, Rocci et al. found no significant difference in half-life between pediatric nephrotic syndrome patients in remission and asthmatic controls. However, in the active phase of disease, the nephrotic syndrome patients did show increased *T*_1/2_ values, which may be explained by the larger volume of distribution in active disease [34]. In patients with nephrotic syndrome, total prednisolone clearance increases proportionally to the increased unbound fraction of prednisolone [34, 38, 42] (Fig. 3). Renal excretion of unchanged drugs is approximately 2–5% for prednisone and 11–24% for prednisolone after administration of either one of the drugs [32].

### Summary

In patients with nephrotic syndrome, the unbound fraction of prednisolone increases due to saturable protein binding. Subsequently, this leads to more rapid elimination and an increase in apparent volume of distribution, in the end, leading to a decrease in total drug concentrations and no change in the steady-state unbound (pharmacologically active) concentration (Fig. 3). Therefore, dose adjustment of prednisone/prednisolone is not necessary in nephrotic syndrome patients with normal renal clearance.

## Pharmacodynamics

Pharmacodynamics refers to what the drug does to the body, including the time course and intensity of therapeutic and adverse effects.

### Therapeutic effects

Clinical efficacy depends on both pharmacokinetic (e.g., what the body does to a drug) and pharmacodynamic (e.g., what the drug does to the body) characteristics of a drug. In case of glucocorticoids, PD may vary greatly among different glucocorticoids, diseases, and individuals. These differences may be explained by a variety of factors: different numbers of GRs per cell, a different glucocorticoid binding affinity, GR diversity, regulatory factors that control gene translation and protein production, and possibly also, differences in non-genomic mechanisms between cell types [54]. One way of comparing drug potency is by the concentration at which 50% of the maximum effect (EC_50_) is achieved. Furthermore, potency is also dependent on the effect monitored. Potential biomarkers for glucocorticoids are endogenous cortisol, T helper and T suppressor lymphocytes, and neutrophil count [55].

### Adverse effects

Prednisolone and prednisone therapy have been associated with a broad range of toxicities. Adverse effects are more common in patients receiving glucocorticoids in high doses or over a long period of time. Potential adverse effects include skin fragility, bodyweight gain, increased risk of infections, and fractures. Important cardiovascular and metabolic effects are hypertension, hyperglycemia, and dyslipidemia [48]. Whereas most anti-inflammatory effects of glucocorticoids are consequences of transrepression of pro-inflammatory and immune genes, adverse events appear to largely result from transactivation that leads to increased expression of regulatory and anti-inflammatory proteins [25, 27, 56].

### Nephrotic syndrome

As it stands, it is not completely understood how prednisolone achieves remission of nephrotic syndrome. High variability exists between individuals with nephrotic syndrome regarding both the efficacy and side effects of prednisone/prednisolone [57]. Furthermore, the molecular basis for the development of clinical resistance to glucocorticoid therapy is unclear in patients with nephrotic syndrome. Inter-individual differences in glucocorticoid handling and metabolism may partly explain the variability in the response to prednisone/prednisolone treatment. However, as previously described in the introduction, differences in disease histology, podocytes, and immunological characteristics of the individual patient may also play a significant role. Different hypotheses exist to explain the mechanism of action of prednisone/prednisolone in patients with nephrotic syndrome. These hypotheses go beyond the conventional anti-inflammatory or immunosuppressive actions, as it is unlikely that the effect is completely due to conventional anti-inflammatory effects of these drugs, since glomerular inflammation is mostly absent in steroid-sensitive nephrotic syndrome.

Previous research has indicated that glomerular podocytes may be a direct target of glucocorticoids in patients with nephrotic syndrome as human podocytes express GRs [58]. The beneficial effect of glucocorticoids might be due to direct protection of podocytes from injury and/or promotion of podocyte repair. Xing et al. showed that dexamethasone upregulated the expression of nephrin [59]. Nephrin is a key component of the slit diaphragm, the main site of control of glomerular permeability. This has resulted in the hypothesis that glucocorticoids act directly on podocytes via promotion of repair with enhanced process formation and upregulation of nephrin. Furthermore, podocyte foot processes consist of cortical actin filaments and actin-associated proteins, which ensure the dynamic maintenance and reorganization of the cytoskeleton. In vitro studies have shown the direct effects of glucocorticoids on podocytes by protection of cultured podocytes via actin filament stabilization and prevention of apoptosis [60, 61]. The effect on apoptosis [61] and upregulation of nephrin [59] appeared to be dose-dependent, which might be an explanation for the observed differences in clinical response to glucocorticoids. Another in vitro study, performed by Guess et al., demonstrated functional glucocorticoid signaling by multiple glucocorticoid-induced responses, including downregulation of the GR [62].

Gamal et al. reported that glomerular GR expression was significantly higher in minimal change early responders in comparison to late responders. Furthermore, a significantly lower glomerular GR expression was found in patients with a steroid-resistant nephrotic syndrome compared to early responders and late responders. Therefore, evaluation of glomerular GR expression at the time of diagnosis can aid in prediction of the response to steroid therapy. This way, exposure to ineffective treatment courses may be prevented in children with nephrotic syndrome [63]. Unfortunately, this technique requires a kidney biopsy and, in daily clinical practice, a kidney biopsy is generally not performed at the time of diagnosis in children with nephrotic syndrome. Therefore, additional approaches are needed to predict the response to steroid therapy, of which pharmacogenetics may be a promising option.

## Pharmacogenetics

It is well known that different patients respond in different ways to the same medication. Many non-genetic factors influence the individual differences in drug response, including age, sex, disease, organ function, concomitant therapy, drug adherence, and drug interactions (for a review on such factors, see [20, 48]). In addition, genetic factors may also have a major influence on the efficacy of a drug and risk of side effects [18, 64]. Pharmacogenetics is the study of the role of inheritance in inter-individual variation in drug response. Genetic factors influencing the patient pharmacokinetic or pharmacodynamic profiles may account for 20–95% of variability in the efficacy and side effects of therapeutic agents [17]. For example, polymorphisms in the CYP3A5 gene account for 40–50% of the variability in tacrolimus dose requirement in Caucasians [65]. After administration, the drug is absorbed and distributed to the site of action. It interacts with targets (such as receptors and enzymes), undergoes metabolism, and is then excreted. Each of these processes could potentially involve a clinical significant genetic variation [18]. Understanding the basis of such variations, i.e., pharmacogenetics, is vital to come to personalized medicine, which ultimately may lead to individualized drug therapy to maximize drug efficacy and minimize drug toxicity.

### Clinical practice

In children with nephrotic syndrome, large inter-individual variability is present in the course of disease, and efficacy and side effects of glucocorticoids. As the variable response to glucocorticoids in patients with nephrotic syndrome cannot completely be attributed to the disease histology, it is difficult to predict the response based on clinical observations alone. For nephrotic syndrome, research on the impact of genetic polymorphisms on steroid response and susceptibility to steroid-related toxicities is limited [19]. For a few other diseases, however, pharmacogenetics has already been implemented in clinical practice [66, 67]. Furthermore, in the field of pediatric nephrology, new guidelines on tacrolimus dosing recommend involvement of CYP3A5 genotyping to optimize the immunosuppressive treatment of the individual transplant patients [68].

In line with the aforementioned examples, we believe that the involvement of pharmacogenetics in the work-up of nephrotic syndrome patients as well might be beneficial, preventing exposure to ineffective drug courses and minimizing drug toxicity. As the mechanism of action of glucocorticoids involves numerous receptors, enzymes, and proteins, a variety of potential targets of genetic polymorphisms may be present. Although limited evidence is available, an overview of previously conducted studies on pharmacogenetics of prednisone and prednisolone in patients with nephrotic syndrome is provided below. Table 1 (glucocorticoid targets) and Table 2 (glucocorticoid PK) provide an overview and brief description of the most important studies conducted in pediatric patients with nephrotic syndrome in which a positive association between the polymorphism and steroid response was described. A summary of the mechanisms and most important results is provided for the targets involved.

**Table 1:** Effects of gene polymorphisms affecting glucocorticoid targets in nephrotic syndrome patients

Target	Gene	(Proposed) mechanism	Population nephrotic syndrome	Polymorphism	Clinical relevance	Ref.
GR	NR3C1	Alteration of GR/GC complex formation	*N* = 108 Age 4.0 (3.1–6.5)	GR-9β + TthIII-1 rs6198 + rs10052957	Higher incidence of glucocorticoid dependence	[69]
			*N* = 118 Age 5.1 (± 3.2)	GTA haplotype rs33388 rs33389 Bcl-1	Higher glucocorticoid sensitivity	[70]
			*N* = 100 Children	rs41423247	Higher incidence of frequent relapsing nephrotic syndrome	[71]
			*N* = 154 Unknown	rs6196 rs10052957 rs258751	Decreased risk of glucocorticoid resistance	[72]
GR heterocomplex	FKBP5	Alteration of GR activity	*N* = 66 Children	rs4713916	Higher incidence of glucocorticoid dependence	[73]
Anti-inflammatory factors	IL-4 promoter	IL-4 production is upregulated in patients with nephrotic syndrome	*N* = 58 Children	rs2243250	Higher frequency in patients with nephrotic syndrome	[74]
			*N* = 150 Age 11.0 (± 6.6)	rs2243250	Association with glucocorticoid resistance	[75]
			*N* = 150 Children	rs2243250	Association with glucocorticoid resistance	[76]
	IL-4Rα		*N* = 85 Children	rs1805010	Lower frequency in patients with frequent relapsing nephrotic syndrome	[77]
	IL-6	IL-6 production is increased in patients with steroid-resistant nephrotic syndrome	*N* = 150 Age 11.0 (± 6.6)	rs1800795	Association with glucocorticoid resistance	[75]
			*N* = 150 Children	rs1800795	Association with glucocorticoid resistance	[76]
Pro-inflammatory factors	IL-12Bpro1	Decrease in IL-12 production	*N* = 79 Age 10.7 (± 4.5)	rs17860508	Association with glucocorticoid dependence	[78]
	TNF-α	Increased TNF transcription, leading to an increase in TNF-α synthesis	*N* = 150 Age 11.0 (± 6.6)	rs1800629	Association with glucocorticoid resistance	[75]
			*N* = 150 Children	rs1800629	Association with glucocorticoid resistance	[76]
	MIF	Increase of MIF level in serum causes a pro-inflammatory response	*N* = 214 Age 3.5 (± 2.9)	rs755622	Association with glucocorticoid resistance	[79]
			*N* = 257 Age 5.8 (± 4.2)	rs755622	Association with glucocorticoid resistance	[80]
			*N* = 80 Children	rs755622	Association with glucocorticoid resistance	[81]

*IL* interleukin; *FKBP5* FK506 binding protein; *MIF* macrophage migration inhibitory factor; *NR3C1* nuclear receptor subfamily 3 group C, member 1; *TNF-α* tumor necrosis factor alpha

**Table 2:** Effects of gene polymorphisms affecting glucocorticoid pharmacokinetics in nephrotic syndrome patients

Target	Gene	(Proposed) mechanism	Population nephrotic syndrome	Polymorphism	Clinical relevance	Ref.
P-glycoprotein	MDR-1	Enhanced P-glycoprotein function	*N* = 108 Age 11.13 (± 4.83)	rs1128503 rs2032582 rs1045642	Association with late response to glucocorticoids	[82]
			*N* = 74 Children	rs1128503	Association with glucocorticoid resistance	[83]
			*N* = 138 Age 4.2 (± 1.6)	rs2032582	Association with glucocorticoid resistance	[84]
			*N* = 216 Children	rs2032582	Association with glucocorticoid resistance	[85]
			*N* = 120 Children	rs1128503	Association with glucocorticoid resistance	[86]
			*N* = 100 Children	rs2032582 rs1128503 rs1045642	Association with different medication regimes	[71]
PXR	NR1I2	Decreased expression of PXR, leading to underexpression of GRs	*N* = 66 Age 4.9 (± 3.7)	rs3842689	Association with glucocorticoid resistance	[87]

*MDR-1* multidrug resistance protein 1, *PXR* pregnane X receptor

### Targets

#### Glucocorticoid receptor

Polymorphisms in the GR gene (NR3C1) are known to be associated with variations in the GR function, because they may alter the formation of the GR/GC complex. Therefore, the hypothesis is that genetic alterations in the gene encoding for the GR receptor may account for some degree of inter-individual variability in the glucocorticoid response and steroid-related toxicity in individuals [88]. Three polymorphisms are known to be associated with reduced sensitivity in both endogenous and exogenous glucocorticoids: TthIIII (rs10052957), ER22/23K (rs6189/rs6190), and GR-9β (rs6198). In contrast, the polymorphisms N363S (rs6195) and BC1I (rs41423247) are associated with an increased sensitivity to glucocorticoids [88, 89]. Increased glucocorticoid sensitivity due to a genetic polymorphism might also be associated with increased susceptibility to steroid-related toxicities. Previously, Eipel et al. showed that pediatric patients with acute lymphoblastic leukemia (ALL) carrying the N363S polymorphism were more prone to steroid-related toxicities [90, 91]. In contrast, children with the ER22/23EK polymorphism were less susceptible [90]. To our knowledge, the role of genetic polymorphisms in the GR gene in susceptibility of steroid-related toxicities has only been investigated in patients with nephrotic syndrome in one study. Teeninga et al. found no association between the GR-9β polymorphism and side effects [69]. To date, a few studies investigated the role of NR3C1 polymorphisms on the glucocorticoid response in pediatric patients with nephrotic syndrome [69–72, 92, 93]. Four studies found a potential influence of genetic polymorphisms in the GR gene on the steroid response in patients with nephrotic syndrome (Table 1).

#### Glucocorticoid receptor heterocomplex

Components of the glucocorticoid heterocomplex are essential to keep the GR in the correct folding for hormone binding and prevent nuclear localization of unoccupied GRs. Abnormalities in the chaperones and co-chaperones that make up the heterocomplex may contribute to decreased glucocorticoid responsiveness, as the integrity of the GR heterocomplex is required for optimal ligand binding and subsequent activation of the transcriptional response. For several diseases, which are treated with glucocorticoids, including nephrotic syndrome, altered levels of chaperone protein hsp90 were found in peripheral blood mononuclear cells from individuals with a steroid-resistant course of disease [94–96]. Although limited, some evidence exists about the association of polymorphisms in the gene encoding for one of the co-chaperones, FKBP5, with steroid resistance in Crohn’s disease [97]. Interestingly, this could also hold true for nephrotic syndrome patients as well. Recently, one study was published on the potential role of FKBP5 polymorphism (rs4713916) in a small group of pediatric nephrotic syndrome patients showing a higher frequency in patients with a steroid-dependent nephrotic syndrome [73].

#### Nuclear translocation receptors

Nuclear translocation receptors, known as importins, play a significant role in the mechanism of glucocorticoid action. These receptors are responsible for the effective transport of the GR/GC complex to the cell nucleus. IPO13 is a primary regulator to facilitate the transfer of the GR/GC complex across the nuclear membrane. In children with asthma, polymorphisms encoding the IPO13 gene resulted in increased sensitivity for glucocorticoids, which was most likely due to the increased availability of glucocorticoids in the nucleus [98]. The role of genetic polymorphisms in the gene encoding for IPO13 in patients with nephrotic syndrome is unknown [19].

#### Pro- and anti-inflammatory factors

To date, the exact underlying pathophysiological mechanisms of nephrotic syndrome are still unknown. One of the hypotheses is that nephrotic syndrome is associated with an immunoregulatory imbalance between T helper subtype 1 (Th1) and T helper subtype 2 (Th2) cells. Cytokines produced by the T helper cells play a role as mediators of inflammation. Several studies have been conducted in patients with various diseases to evaluate the association with genetic polymorphisms in the IL-1, IL-4, IL-6, IL-13, and TNF-α genes. The evidence for genetic polymorphisms in the cytokine genes in patients with nephrotic syndrome is, however, limited.

Minimal change nephrotic syndrome is associated with atopy and IgE production [99]. T helper subtype 2 cytokines, such as IL-4 and IL-13, are known to be involved in the development of atopy. Previously genetic variations of IL-4 and IL-13 and their receptors have been shown to be associated with predisposition to atopy and/or elevated serum IgE levels [100]. Several studies have been conducted to investigate the role of polymorphisms in the genes coding for IL-4, IL-6, and IL-13 in pediatric patients with nephrotic syndrome [74–76, 101–103]. The IL-4 polymorphism rs2243250 was associated with nephrotic syndrome and an increased risk of steroid resistance [74–76]. Furthermore, previous research conducted by Jafar et al. and Tripathi et al. indicate that a genetic polymorphism in the IL-6 gene is associated with decreased responsiveness to steroids [75, 76]. No significant association was found between the IL-13 gene polymorphisms and disease susceptibility or steroid responsiveness [71, 101, 103].

An important pro-inflammatory cytokine is macrophage migration inhibitory factor (MIF). MIF has the unique ability to override the inhibitory effects of glucocorticoids on the immune system. Due to its regulatory properties, MIF is considered a critical mediator in various immune and inflammatory diseases. The allele MIF-173*C (rs755622) is associated with higher serum MIF levels. Several studies have been conducted to investigate the potential role of this genetic polymorphism in the gene encoding for MIF in patients with nephrotic syndrome [71, 79–81, 104, 105]. A meta-analysis conducted by Tong et al. showed that the gene polymorphism rs755622 plays an important role in the risk of glucocorticoid resistance in patients with nephrotic syndrome [106]. The hypothesis is that the G/C substitution at 173 bp of the MIF gene increases the MIF level in serum and could therefore cause a pro-inflammatory response, induce injury to podocytes, and accelerate the progression of glomerulosclerosis [107]. TNF-α is also an important pro-inflammatory cytokine involved in the inflammatory process. Elevation of TNF-α has been found in the plasma and urine of patients with nephrotic syndrome [108, 109]. Conflicting results have been published for the role polymorphisms in the gene encoding for TNF-α in patients with nephrotic syndrome [75, 76, 110, 111]. Lastly, Müller-Berghaus et al. investigated the role of polymorphisms in gene encoding for pro-inflammatory mediator IL-12B and found an association of the IL12Bpro-1.1 genotype with a steroid-dependent course of disease [78].

#### GLCCI1 (glucocorticoid-induced transcript 1 gene)

Little is known about the exact function of GLCCI1. GLCCI1 was initially described as a thymocyte-specific transcript that is rapidly upregulated in response to dexamethasone treatment [112]. In addition, GLCCI1 is expressed in the kidney and, in particular, in the glomeruli. Knockdown of the GLCCI1 gene resulted in disruption of the glomerular permeability filter and podocyte foot process effacement. A genome-wide association study in patients with asthma showed a significant association between the genetic polymorphism rs37972 of the GLCCI1 gene and a decreased response to glucocorticoid inhalation therapy [113]. In contrast, two studies in pediatric nephrotic syndrome patients could not confirm the association between this specific polymorphism and steroid responsiveness in patients with nephrotic syndrome [71, 114].

#### P-glycoprotein

P-glycoprotein is an efflux pump encoded by the multidrug resistance protein 1 gene (MDR1). Glucocorticoids are known substrates for P-glycoprotein and may also induce P-glycoprotein expression [52, 115]. In the kidney, P-glycoprotein is expressed in the brush border membrane of proximal tubular epithelial cells. Increased expression of P-glycoprotein results in decreased intracellular drug concentrations and may consequently decrease treatment response. Previous research has shown higher expression of MDR1 and increased P-glycoprotein activity in children with steroid-resistant nephrotic syndrome [116, 117]. To date, approximately 50 genetic polymorphisms have been reported in the MDR1 gene. Among the genetic polymorphisms, C1236T (rs1128503), G2677T/A (rs2032582), and C3435T (rs1045642) are the most common variants in the coding region of MDR1. The interpretation of the influence of the genetic polymorphisms on P-glycoprotein expression, however, is unresolved and may vary depending on tissue type, pathological status, and ethnicity [118]. A recent systematic review on pharmacogenetics and adverse drug reactions in pediatric oncology patients indicated protective effects from two genetic polymorphisms of the MDR1 gene in methotrexate- and vincristine-related neurotoxicity in pediatric ALL patients [119]. In nephrotic syndrome patients, however, no studies have been conducted to investigate the potential role of genetic polymorphisms in the MDR1 gene in steroid-related toxicities. Several studies have been conducted to evaluate the association of P-glycoprotein polymorphisms with the responsiveness to glucocorticoids in patients with nephrotic syndrome. The results of these studies on the significance of the genetic polymorphisms are contradictory [71, 82–84, 86, 104, 120, 121]. A recent meta-analysis concluded that there is evidence of an association between rs1128503 and increased risk of steroid resistance in children with nephrotic syndrome [122].

#### Pregnane X receptor

Pregnane X receptor (PXR) gene (NR1I2) encodes an intracellular receptor that, upon binding with glucocorticoids or xenobiotic substances, activates a set of genes involved in the metabolism of drugs. Turolo et al. described an association of the presence of a PXR deletion polymorphism (rs3842689) with steroid resistance. The hypothesis is that a reduced expression of PXR leads to an underexpression of GRs, which may be the explanation for the development of steroid resistance [87].

### Summary

The results of the aforementioned reported papers are generally inconclusive and contradictory. However, some genetic polymorphisms appear to be promising in the prediction of steroid response or steroid-related toxicities in children with nephrotic syndrome. Especially, polymorphisms in the genes encoding for the GR and GR heterocomplex seem to have an association with steroid responsiveness. Nevertheless, most studies are hampered by small patient cohorts. Therefore, studies in larger cohorts with nephrotic syndrome patients are necessary to draw conclusions about the influence of genetic polymorphisms on the glucocorticoid response. Furthermore, as mentioned above, pharmacogenetics may also play a role in the intensity and spectrum of side effects. Currently, little is known about the influence of pharmacogenetics on steroid-related toxicities in patients with nephrotic syndrome. However, as previous research in mostly cancer patients has shown a potential role of genetic polymorphisms in the susceptibility on steroid-related toxicities, this area is an important opportunity for future research as well.

## Conclusion

Glucocorticoids are essential in the treatment of childhood nephrotic syndrome. Currently, standardized treatment guidelines with high doses of prednisone or prednisolone are proposed worldwide. As current treatment guidelines are largely based on empiric recommendations rather than clinical trials, large variability in the treatment of nephrotic syndrome is present among physicians [4], especially regarding the treatment of subsequent relapses and the choice of second-line immunosuppressive drugs. As large-scale clinical trials are lacking, treatment decisions are frequently based on either the preference or common practice of the treating physician or guidelines of the country, rather than the individual characteristics of the patient. Therefore, effort should be made to first provide international guidelines based on clinical trials to uniformly treat patients with nephrotic syndrome. Subsequently, effort should be made to identify specific markers to individualize treatment, as large inter-individual differences are present in both the clinical course of disease and adverse effects of glucocorticoids in children with nephrotic syndrome. Pharmacogenetics has a promising role in working towards personalized medicine. Despite the fact that the evidence about the role of pharmacogenetics in children with nephrotic syndrome is limited, we feel that available data do show a potential role for pharmacogenetics in clinical practice to maximize drug efficacy, minimize drug toxicity, and avoid exposure to ineffective drug courses. Nowadays, the evidence to implement these genetic markers in clinical practice is too little and, therefore, clinical implementation of pharmacogenetics in nephrotic syndrome patients is not possible yet. Therefore, we feel that further research is highly important to identify specific and sensitive markers for steroid resistance in patients without genetic podocyte mutations as well as for patients more at risk for steroid-related toxicities. As nephrotic syndrome is a rare kidney disease in childhood and large patient cohorts are needed to ultimately implement pharmacogenetics in the clinical work-up, we believe that this research preferably should be conducted in international collaborative studies.

## Multiple choice questions (answers are provided following the reference list)

1. Current glucocorticoid dosing guidelines for the treatment of nephrotic syndrome are
  - Standardized
  - Individualized
  - Based on randomized controlled trials
2. The genomic glucocorticoid action is characterized by
  - A rapid onset of the effect
  - Short duration of the effect
  - A slow onset of the effect
3. Adverse events of prednisone/prednisolone largely result from
  - Transrepression of pro-inflammatory and immune genes
  - Transactivation of anti-inflammatory genes
4. Due to decreased protein binding of prednisone and prednisolone in patients with nephrotic syndrome and to more rapid elimination and an increase in volume of distribution, the steady-state unbound concentration
  - is increased
  - is unchanged
  - is decreased
5. Pharmacogenetics may have an influence on the profile of the individual patient
  - Pharmacokinetic
  - Pharmacodynamic
  - Pharmacokinetic and pharmacodynamic
  - None of the above

## References

[1] El Bakkali L, Rodrigues Pereira R, Kuik DJ, Ket JC, van Wijk JA (2011). Nephrotic syndrome in the Netherlands: a population-based cohort study and a review of the literature. Pediatr Nephrol.

[2] Tarshish P, Tobin JN, Bernstein J, Edelmann CM (1997). Prognostic significance of the early course of minimal change nephrotic syndrome: report of the international study of kidney disease in children. J Am Soc Nephrol.

[3] Kidney Disease: Improving Global Outcomes (KDIGO) Glomerulonephritis Work Group (2012) KDIGO clinical practice guideline for glomerulonephritis. Kidney Int Suppl 2:139–274

[4] Samuel SM, Flynn R, Zappitelli M, Dart A, Parekh R, Pinsk M, Mammen C, Wade A, Scott SD, Canadian Childhood Nephrotic Syndrome Project T (2017). Factors influencing practice variation in the management of nephrotic syndrome: a qualitative study of pediatric nephrology care providers. CMAJ Open.

[5] Teeninga N, Kist-van Holthe JE, van Rijswijk N, de Mos NI, Hop WC, Wetzels JF, van der Heijden AJ, Nauta J (2013). Extending prednisolone treatment does not reduce relapses in childhood nephrotic syndrome. J Am Soc Nephrol.

[6] Webb N, Wooley R, Brettell E, Cummins C, Trompeter R, Barsoum E, Ives N (2017) Standard vs. extended course prednisolone therapy for the presenting episode of steroid sensitive nephrotic syndrome: the PREDNOS study. (abstract) ESPN 50th annual meeting

[7] Schijvens AM, Dorresteijn EM, Roeleveld N, Ter Heine R, van Wijk JAE, Bouts AHM, Keijzer-Veen MG, van de Kar N, van den Heuvel L, Schreuder MF (2017). REducing STEroids in Relapsing Nephrotic syndrome: the RESTERN study—protocol of a national, double-blind, randomised, placebo-controlled, non-inferiority intervention study. BMJ Open.

[8] Saleem MA (2015). One hundred ways to kill a podocyte. Nephrol Dial Transplant.

[9] Vivarelli M, Massella L, Ruggiero B, Emma F (2017). Minimal change disease. Clin J Am Soc Nephrol.

[10] Bierzynska A, McCarthy HJ, Soderquest K, Sen ES, Colby E, Ding WY, Nabhan MM, Kerecuk L, Hegde S, Hughes D, Marks S, Feather S, Jones C, Webb NJ, Ognjanovic M, Christian M, Gilbert RD, Sinha MD, Lord GM, Simpson M, Koziell AB, Welsh GI, Saleem MA (2017). Genomic and clinical profiling of a national nephrotic syndrome cohort advocates a precision medicine approach to disease management. Kidney Int.

[11] Gallon L, Leventhal J, Skaro A, Kanwar Y, Alvarado A (2012). Resolution of recurrent focal segmental glomerulosclerosis after retransplantation. N Engl J Med.

[12] Bierzynska A, Saleem M (2017). Recent advances in understanding and treating nephrotic syndrome. F1000Res.

[13] Wada T, Nangaku M (2015). A circulating permeability factor in focal segmental glomerulosclerosis: the hunt continues. Clin Kidney J.

[14] Elie V, Fakhoury M, Deschenes G, Jacqz-Aigrain E (2012). Physiopathology of idiopathic nephrotic syndrome: lessons from glucocorticoids and epigenetic perspectives. Pediatr Nephrol.

[15] (1978) Nephrotic syndrome in children: prediction of histopathology from clinical and laboratory characteristics at time of diagnosis. A report of the International Study of Kidney Disease in Children. Kidney Int 13:159–165 10.1038/ki.1978.23 713276

[16] Weinshilboum R, Wang L (2004). Pharmacogenomics: bench to bedside. Nat Rev Drug Discov.

[17] Evans WE, McLeod HL (2003). Pharmacogenomics—drug disposition, drug targets, and side effects. N Engl J Med.

[18] Weinshilboum R (2003). Inheritance and drug response. N Engl J Med.

[19] Cuzzoni E, De Iudicibus S, Franca R, Stocco G, Lucafo M, Pelin M, Favretto D, Pasini A, Montini G, Decorti G (2015). Glucocorticoid pharmacogenetics in pediatric idiopathic nephrotic syndrome. Pharmacogenomics.

[20] Czock D, Keller F, Rasche FM, Haussler U (2005). Pharmacokinetics and pharmacodynamics of systemically administered glucocorticoids. Clin Pharmacokinet.

[21] Ponec M, Kempenaar J, Shroot B, Caron JC (1986). Glucocorticoids: binding affinity and lipophilicity. J Pharm Sci.

[22] Kirschke E, Goswami D, Southworth D, Griffin PR, Agard DA (2014). Glucocorticoid receptor function regulated by coordinated action of the Hsp90 and Hsp70 chaperone cycles. Cell.

[23] Payne DN, Adcock IM (2001). Molecular mechanisms of corticosteroid actions. Paediatr Respir Rev.

[24] Barnes PJ (2010). Mechanisms and resistance in glucocorticoid control of inflammation. J Steroid Biochem Mol Biol.

[25] Adcock IM (2000). Molecular mechanisms of glucocorticosteroid actions. Pulm Pharmacol Ther.

[26] Kagoshima M, Ito K, Cosio B, Adcock IM (2003). Glucocorticoid suppression of nuclear factor-kappa B: a role for histone modifications. Biochem Soc Trans.

[27] Lowenberg M, Stahn C, Hommes DW, Buttgereit F (2008). Novel insights into mechanisms of glucocorticoid action and the development of new glucocorticoid receptor ligands. Steroids.

[28] Alangari AA (2010). Genomic and non-genomic actions of glucocorticoids in asthma. Ann Thorac Med.

[29] Croxtall JD, Choudhury Q, Flower RJ (2000). Glucocorticoids act within minutes to inhibit recruitment of signalling factors to activated EGF receptors through a receptor-dependent, transcription-independent mechanism. Br J Pharmacol.

[30] Rostin M, Barthe P, Houin G, Alvinerie M, Bouissou F (1990). Pharmacokinetics of prednisolone in children with the nephrotic syndrome. Pediatr Nephrol.

[31] Gatti G, Perucca E, Frigo GM, Notarangelo LD, Barberis L, Martini A (1984). Pharmacokinetics of prednisone and its metabolite prednisolone in children with nephrotic syndrome during the active phase and in remission. Br J Clin Pharmacol.

[32] Rose JQ, Yurchak AM, Jusko WJ (1981). Dose dependent pharmacokinetics of prednisone and prednisolone in man. J Pharmacokinet Biopharm.

[33] Green OC, Winter RJ, Kawahara FS, Phillips LS, Lewy PR, Hart RL, Pachman LM (1978). Plasma levels, half-life values, and correlation with physiologic assays for growth and immunity. J Pediatr.

[34] Rocci ML, Assael BM, Appiani AC, Edefonti A, Jusko WJ (1982). Effect on nephrotic syndrome on absorption and disposition of prednisolone in children. Int J Pediatr Nephrol.

[35] Frey BM, Frey FJ (1990). Clinical pharmacokinetics of prednisone and prednisolone. Clin Pharmacokinet.

[36] Pickup ME (1979). Clinical pharmacokinetics of prednisone and prednisolone. Clin Pharmacokinet.

[37] Frey FJ, Frey BM (1984). Altered plasma protein-binding of prednisolone in patients with the nephrotic syndrome. Am J Kidney Dis.

[38] Frey FJ, Frey BM (1982). Altered prednisolone kinetics in patients with the nephrotic syndrome. Nephron.

[39] Bennett WM, Aronoff GR, Golper TA, Pulliam J, Wolfson M, Singer I (1994). Drug prescribing in renal failure.

[40] Miller PF, Bowmer CJ, Wheeldon J, Brocklebank JT (1990). Pharmacokinetics of prednisolone in children with nephrosis. Arch Dis Child.

[41] Teeninga N, Guan Z, Stevens J, Kist-van Holthe JE, Ackermans MT, van der Heijden AJ, van Schaik RH, van Gelder T, Nauta J (2016). Population pharmacokinetics of prednisolone in relation to clinical outcome in children with nephrotic syndrome. Ther Drug Monit.

[42] Bergrem H (1983). Pharmacokinetics and protein binding of prednisolone in patients with nephrotic syndrome and patients undergoing hemodialysis. Kidney Int.

[43] Gugler R, Shoeman DW, Huffman DH, Cohlmia JB, Azarnoff DL (1975). Pharmacokinetics of drugs in patients with the nephrotic syndrome. J Clin Invest.

[44] Diederich S, Eigendorff E, Burkhardt P, Quinkler M, Bumke-Vogt C, Rochel M, Seidelmann D, Esperling P, Oelkers W, Bahr V (2002). 11beta-hydroxysteroid dehydrogenase types 1 and 2: an important pharmacokinetic determinant for the activity of synthetic mineralo- and glucocorticoids. J Clin Endocrinol Metab.

[45] Zurcher RM, Frey BM, Frey FJ (1989). Impact of ketoconazole on the metabolism of prednisolone. Clin Pharmacol Ther.

[46] Lee KH, Shin JG, Chong WS, Kim S, Lee JS, Jang IJ, Shin SG (1993). Time course of the changes in prednisolone pharmacokinetics after co-administration or discontinuation of rifampin. Eur J Clin Pharmacol.

[47] Bartoszek M, Brenner AM, Szefler SJ (1987). Prednisolone and methylprednisolone kinetics in children receiving anticonvulsant therapy. Clin Pharmacol Ther.

[48] Bergmann TK, Barraclough KA, Lee KJ, Staatz CE (2012). Clinical pharmacokinetics and pharmacodynamics of prednisolone and prednisone in solid organ transplantation. Clin Pharmacokinet.

[49] Frey FJ, Schnetzer A, Horber FF, Frey BM (1987). Evidence that cyclosporine does not affect the metabolism of prednisolone after renal transplantation. Transplantation.

[50] Ekka BK, Bagga A, Srivastava RN (1997). Single- versus divided-dose prednisolone therapy for relapses of nephrotic syndrome. Pediatr Nephrol.

[51] Usui T, Saitoh Y, Komada F (2003). Induction of CYP3As in HepG2 cells by several drugs. Association between induction of CYP3A4 and expression of glucocorticoid receptor. Biol Pharm Bull.

[52] Shimada T, Terada A, Yokogawa K, Kaneko H, Nomura M, Kaji K, Kaneko S, Kobayashi K, Miyamoto K (2002). Lowered blood concentration of tacrolimus and its recovery with changes in expression of CYP3A and P-glycoprotein after high-dose steroid therapy. Transplantation.

[53] Amin ML (2013). P-glycoprotein inhibition for optimal drug delivery. Drug Target Insights.

[54] Bamberger CM, Schulte HM, Chrousos GP (1996). Molecular determinants of glucocorticoid receptor function and tissue sensitivity to glucocorticoids. Endocr Rev.

[55] Cuzzoni E, De Iudicibus S, Stocco G, Favretto D, Pelin M, Messina G, Ghio L, Monti E, Pasini A, Montini G, Decorti G (2016). In vitro sensitivity to methyl-prednisolone is associated with clinical response in pediatric idiopathic nephrotic syndrome. Clini Pharm Ther.

[56] Stahn C, Lowenberg M, Hommes DW, Buttgereit F (2007). Molecular mechanisms of glucocorticoid action and selective glucocorticoid receptor agonists. Mol Cell Endocrinol.

[57] Eddy AA, Symons JM (2003). Nephrotic syndrome in childhood. Lancet.

[58] Yan K, Kudo A, Hirano H, Watanabe T, Tasaka T, Kataoka S, Nakajima N, Nishibori Y, Shibata T, Kohsaka T, Higashihara E, Tanaka H, Watanabe H, Nagasawa T, Awa S (1999). Subcellular localization of glucocorticoid receptor protein in the human kidney glomerulus. Kidney Int.

[59] Xing CY, Saleem MA, Coward RJ, Ni L, Witherden IR, Mathieson PW (2006). Direct effects of dexamethasone on human podocytes. Kidney Int.

[60] Ransom RF, Lam NG, Hallett MA, Atkinson SJ, Smoyer WE (2005). Glucocorticoids protect and enhance recovery of cultured murine podocytes via actin filament stabilization. Kidney Int.

[61] Wada T, Pippin JW, Marshall CB, Griffin SV, Shankland SJ (2005). Dexamethasone prevents podocyte apoptosis induced by puromycin aminonucleoside: role of p53 and Bcl-2-related family proteins. J Am Soc Nephrol.

[62] Guess A, Agrawal S, Wei CC, Ransom RF, Benndorf R, Smoyer WE (2010). Dose- and time-dependent glucocorticoid receptor signaling in podocytes. Am J Physiol - Renal Physiol.

[63] Gamal Y, Badawy A, Swelam S, Tawfeek MS, Gad EF (2017). Glomerular glucocorticoid receptors expression and clinicopathological types of childhood nephrotic syndrome. Fetal Pediatr Pathol.

[64] Evans WE (2003). Pharmacogenomics: marshalling the human genome to individualise drug therapy. Gut.

[65] Andrews LM, Li Y, De Winter BCM, Shi YY, Baan CC, Van Gelder T, Hesselink DA (2017). Pharmacokinetic considerations related to therapeutic drug monitoring of tacrolimus in kidney transplant patients. Expert Opin Drug Metab Toxicol.

[66] Johnson JA (2013). Pharmacogenetics in clinical practice: how far have we come and where are we going?. Pharmacogenomics.

[67] Nelson MR, Johnson T, Warren L, Hughes AR, Chissoe SL, Xu CF, Waterworth DM (2016). The genetics of drug efficacy: opportunities and challenges. Nat Rev Genet.

[68] Birdwell KA, Decker B, Barbarino JM, Peterson JF, Stein CM, Sadee W, Wang D, Vinks AA, He Y, Swen JJ, Leeder JS, van Schaik R, Thummel KE, Klein TE, Caudle KE, MacPhee IA (2015). Clinical Pharmacogenetics Implementation Consortium (CPIC) guidelines for CYP3A5 genotype and tacrolimus dosing. Clin Pharmacol Ther.

[69] Teeninga N, Kist-van Holthe JE, van den Akker EL, Kersten MC, Boersma E, Krabbe HG, Knoers NV, van der Heijden AJ, Koper JW, Nauta J (2014). Genetic and in vivo determinants of glucocorticoid sensitivity in relation to clinical outcome of childhood nephrotic syndrome. Kidney Int.

[70] Zalewski G, Wasilewska A, Zoch-Zwierz W, Chyczewski L (2008). Response to prednisone in relation to NR3C1 intron B polymorphisms in childhood nephrotic syndrome. Pediatr Nephrol.

[71] Suvanto M, Jahnukainen T, Kestila M, Jalanko H (2016). Single nucleotide polymorphisms in pediatric idiopathic nephrotic syndrome. Int J Nephrol.

[72] Liu J, Wan Z, Song Q, Li Z, He Y, Tang Y, Xie W, Xie Y, Zhang J (2017) NR3C1 gene polymorphisms are associated with steroid resistance in patients with primary nephrotic syndrome. Pharmacogenomics. 10.2217/pgs-2017-0084 10.2217/pgs-2017-0084 29207898

[73] Du N, Yang F, Li L, Liu X, Sun L, Zhang S, He X, Tang Y, Shi J, Liu C, Zhang X (2017). Association of single-nucleotide polymorphism in the FKBP5 gene with response to steroids in pediatric patients with primary nephrotic syndrome. Clin Nephrol.

[74] Kobayashi Y, Arakawa H, Suzuki M, Takizawa T, Tokuyama K, Morikawa A (2003). Polymorphisms of interleukin-4-related genes in Japanese children with minimal change nephrotic syndrome. Am J Kidney Dis.

[75] Jafar T, Agrawal S, Mahdi AA, Sharma RK, Awasthi S, Agarwal GG (2011). Cytokine gene polymorphism in idiopathic nephrotic syndrome children. Indian J Clin Biochem.

[76] Tripathi G, Jafar T, Mandal K, Mahdi AA, Awasthi S, Sharma RK, Kumar A, Gulati S, Agrawal S (2008). Does cytokine gene polymorphism affect steroid responses in idiopathic nephrotic syndrome?. Indian J Med Sci.

[77] Ikeuchi Y, Kobayashi Y, Arakawa H, Suzuki M, Tamra K, Morikawa A (2009). Polymorphisms in interleukin-4-related genes in patients with minimal change nephrotic syndrome. Pediatr Nephrol.

[78] Müller-Berghaus J, Kemper MJ, Hoppe B, Querfeld U, Müller-Wiefel DE, Morahan G, Schadendorf D, Tenbrock K (2008). The clinical course of steroid-sensitive childhood nephrotic syndrome is associated with a functional IL12B promoter polymorphism. Nephrol Dial Transplant.

[79] Berdeli A, Mir S, Ozkayin N, Serdaroglu E, Tabel Y, Cura A (2005). Association of macrophage migration inhibitory factor -173C allele polymorphism with steroid resistance in children with nephrotic syndrome. Pediatr Nephrol.

[80] Vivarelli M, D’Urbano LE, Stringini G, Ghiggeri GM, Caridi G, Donn R, Tozzi A, Emma F, De Benedetti F (2008). Association of the macrophage migration inhibitory factor -173*C allele with childhood nephrotic syndrome. Pediatr Nephrol.

[81] Ramayani OR, Sekarwana N, Trihono PP, Sadewa AH, Lelo A (2016). A genetic study of steroid-resistant nephrotic syndrome: relationship between polymorphism -173 G to C in the MIF gene and serum level MIF in children. J Dev Orig Health Dis.

[82] Wasilewska A, Zalewski G, Chyczewski L, Zoch-Zwierz W (2007). MDR-1 gene polymorphisms and clinical course of steroid-responsive nephrotic syndrome in children. Pediatr Nephrol.

[83] Chiou YH, Wang LY, Wang TH, Huang SP (2012). Genetic polymorphisms influence the steroid treatment of children with idiopathic nephrotic syndrome. Pediatr Nephrol.

[84] Youssef DM, Attia TA, El-Shal AS, Abduelometty FA (2013). Multi-drug resistance-1 gene polymorphisms in nephrotic syndrome: impact on susceptibility and response to steroids. Gene.

[85] Jafar T, Prasad N, Agarwal V, Mahdi A, Gupta A, Sharma RK, Negi MP, Agrawal S (2011). MDR-1 gene polymorphisms in steroid-responsive versus steroid-resistant nephrotic syndrome in children. Nephrol Dial Transplant.

[86] Safan MA, Elhelbawy NG, Midan DA, Khader HF (2017). ABCB1 polymorphisms and steroid treatment in children with idiopathic nephrotic syndrome. Br J Biomed Sci.

[87] Turolo S, Edefonti A, Lepore M, Ghio L, Cuzzoni E, Decorti G, Pasini A, Materassi M, Malaventura C, Pugliese F, Montini G (2016). SXR rs3842689: a prognostic factor for steroid sensitivity or resistance in pediatric idiopathic nephrotic syndrome. Pharmacogenomics.

[88] van Rossum EF, Lamberts SW (2004). Polymorphisms in the glucocorticoid receptor gene and their associations with metabolic parameters and body composition. Recent Prog Horm Res.

[89] De Iudicibus S, Franca R, Martelossi S, Ventura A, Decorti G (2011). Molecular mechanism of glucocorticoid resistance in inflammatory bowel disease. World J Gastroenterol.

[90] Eipel O, Hegyi M, Csordas K, Nemeth K, Luczay A, Torok D, Csoka M, Erdelyi D, Kovacs G (2016). Some GCR polymorphisms (N363S, ER22/23EK, and Bcl-1) may influence steroid-induced toxicities and survival rates in children with ALL. J Pediatr Hematol Oncol.

[91] Eipel OT, Nemeth K, Torok D, Csordas K, Hegyi M, Ponyi A, Ferenczy A, Erdelyi DJ, Csoka M, Kovacs GT (2013). The glucocorticoid receptor gene polymorphism N363S predisposes to more severe toxic side effects during pediatric acute lymphoblastic leukemia (ALL) therapy. Int J Hematol.

[92] Ye J, Yu Z, Ding J, Chen Y, Huang J, Yao Y, Xiao H, Yang J, Shen Y, Meng Q (2006). Genetic variations of the NR3C1 gene in children with sporadic nephrotic syndrome. Biochem Biophys Res Commun.

[93] Cho HY, Choi HJ, Lee SL, Lee HK, Kang HK, Ha IS, Choi Y, Cheong HI (2009). Polymorphisms of the NR3C1 gene in Korean children with nephrotic syndrome. Korean J Pediatr.

[94] Ouyang J, Chen P, Jiang T, Chen Y, Li J (2012). Nuclear HSP90 regulates the glucocorticoid responsiveness of PBMCs in patients with idiopathic nephrotic syndrome. Int Immunopharmacol.

[95] Qian X, Zhu Y, Xu W, Lin Y (2001). Glucocorticoid receptor and heat shock protein 90 in peripheral blood mononuclear cells from asthmatics. Chin Med J.

[96] Kojika S, Sugita K, Inukai T, Saito M, Iijima K, Tezuka T, Goi K, Shiraishi K, Mori T, Okazaki T, Kagami K, Ohyama K, Nakazawa S (1996). Mechanisms of glucocorticoid resistance in human leukemic cells: implication of abnormal 90 and 70 kDa heat shock proteins. Leukemia.

[97] Maltese P, Palma L, Sfara C, de Rocco P, Latiano A, Palmieri O, Corritore G, Annese V, Magnani M (2012). Glucocorticoid resistance in Crohn’s disease and ulcerative colitis: an association study investigating GR and FKBP5 gene polymorphisms. Pharmacogenomics J.

[98] Raby BA, Van Steen K, Lasky-Su J, Tantisira K, Kaplan F, Weiss ST (2009). Importin-13 genetic variation is associated with improved airway responsiveness in childhood asthma. Respir Res.

[99] Groshong T, Mendelson L, Mendoza S, Bazaral M, Hamburger R, Tune B (1973). Serum IgE in patients with minimal-change nephrotic syndrome. J Pediatr.

[100] Hershey GK, Friedrich MF, Esswein LA, Thomas ML, Chatila TA (1997). The association of atopy with a gain-of-function mutation in the alpha subunit of the interleukin-4 receptor. N Engl J Med.

[101] Tenbrock K, Schubert A, Stapenhorst L, Kemper MJ, Gellermann J, Timmermann K, Müller-Wiefel DE, Querfeld U, Hoppe B, Michalk D (2002). Type I IgE receptor, interleukin 4 receptor and interleukin 13 polymorphisms in children with nephrotic syndrome. Clin Sci (Lond).

[102] Parry RG, Gillespie KM, Parnham A, Clark AG, Mathieson PW (1999). Interleukin-4 and interleukin-4 receptor polymorphisms in minimal change nephropathy. Clin Sci (Lond).

[103] Wei CL, Cheung W, Heng CK, Arty N, Chong SS, Lee BW, Puah KL, Yap HK (2005). Interleukin-13 genetic polymorphisms in Singapore Chinese children correlate with long-term outcome of minimal-change disease. Nephrol Dial Transplant.

[104] Choi HJ, Cho HY, Ro H, Lee SH, Han KH, Lee HK, Kang HG, Ha SI, Choi Y, Cheong HI (2011). Polymorphisms of the MDR1 and MIF genes in children with nephrotic syndrome. Pediatr Nephrol.

[105] Swierczewska M, Ostalska-Nowicka D, Kempisty B, Szczepankiewicz A, Nowicki M (2014). Polymorphic variants of MIF gene and prognosis in steroid therapy in children with idiopathic nephrotic syndrome. Acta Biochim Pol.

[106] Tong X, He J, Liu S, Peng S, Yan Z, Zhang Y, Fan H (2015). Macrophage migration inhibitory factor -173G/C gene polymorphism increases the risk of renal disease: a meta-analysis. Nephrology (Carlton).

[107] Sasaki S, Nishihira J, Ishibashi T, Yamasaki Y, Obikane K, Echigoya M, Sado Y, Ninomiya Y, Kobayashi K (2004). Transgene of MIF induces podocyte injury and progressive mesangial sclerosis in the mouse kidney. Kidney Int.

[108] Suranyi MG, Guasch A, Hall BM, Myers BD (1993). Elevated levels of tumor necrosis factor-alpha in the nephrotic syndrome in humans. Am J Kidney Dis.

[109] Bustos C, Gonzalez E, Muley R, Alonso JL, Egido J (1994). Increase of tumour necrosis factor alpha synthesis and gene expression in peripheral blood mononuclear cells of children with idiopathic nephrotic syndrome. Eur J Clin Investig.

[110] Kim SD, Park JM, Kim IS, Choi KD, Lee BC, Lee SH, Hong SJ, Jin SY, Lee HJ, Hong MS, Chung JH, Lee TW, Ihm CG, Cho BS (2004). Association of IL-1beta, IL-1ra, and TNF-alpha gene polymorphisms in childhood nephrotic syndrome. Pediatr Nephrol.

[111] Tieranu I, Dutescu MI, Bara C, Tieranu CG, Balgradean M, Popa OM (2017). Preliminary study regarding the association between tumor necrosis factor alpha gene polymorphisms and childhood idiopathic nephrotic syndrome in Romanian pediatric patients. Maedica (Buchar).

[112] Chapman MS, Qu N, Pascoe S, Chen WX, Apostol C, Gordon D, Miesfeld RL (1995). Isolation of differentially expressed sequence tags from steroid-responsive cells using mRNA differential display. Mol Cell Endocrinol.

[113] Tantisira KG, Lasky-Su J, Harada M, Murphy A, Litonjua AA, Himes BE, Lange C, Lazarus R, Sylvia J, Klanderman B, Duan QL, Qiu W, Hirota T, Martinez FD, Mauger D, Sorkness C, Szefler S, Lazarus SC, Lemanske RF, Peters SP, Lima JJ, Nakamura Y, Tamari M, Weiss ST (2011). Genomewide association between GLCCI1 and response to glucocorticoid therapy in asthma. N Engl J Med.

[114] Cheong HI, Kang HG, Schlondorff J (2012). GLCCI1 single nucleotide polymorphisms in pediatric nephrotic syndrome. Pediatr Nephrol.

[115] Karssen AM, Meijer OC, van der Sandt IC, De Boer AG, De Lange EC, De Kloet ER (2002). The role of the efflux transporter P-glycoprotein in brain penetration of prednisolone. J Endocrinol.

[116] Wasilewska A, Zoch-Zwierz W, Pietruczuk M, Zalewski G (2006). Expression of P-glycoprotein in lymphocytes from children with nephrotic syndrome, depending on their steroid response. Pediatr Nephrol.

[117] Stachowski J, Zanker CB, Runowski D, Zaniew M, Peszko A, Medynska A, Zwolinska D, Rogowska-Kalisz A, Hyla-Klekot L, Szprygner K, Weglarska J, Sieniawska M, Musial W, Maciejewski J, Baldamus CA (2000). Resistance to therapy in primary nephrotic syndrome: effect of MDR1 gene activity. [Polish] (Odpornosc na leczenie w przebiegu pierwotnego zespolu nerczycowego: wplyw aktywnosci genu MDR1.). Polski merkuriusz lekarski: organ Polskiego Towarzystwa Lekarskiego.

[118] Sakaeda T, Nakamura T, Okumura K (2003). Pharmacogenetics of MDR1 and its impact on the pharmacokinetics and pharmacodynamics of drugs. Pharmacogenomics.

[119] Conyers R, Devaraja S, Elliott D (2017) Systematic review of pharmacogenomics and adverse drug reactions in paediatric oncology patients. Pediatr Blood Cancer. 10.1002/pbc.26937 10.1002/pbc.26937 29286579

[120] Dhandapani MC, Venkatesan V, Rengaswamy NB, Gowrishankar K, Nageswaran P, Perumal V (2015). Association of ACE and MDR1 gene polymorphisms with steroid resistance in children with idiopathic nephrotic syndrome. Genet Test Mol Biomarkers.

[121] Cizmarikova M, Podracka L, Klimcakova L, Habalova V, Boor A, Mojzis J, Mirossay L (2015). MDR1 polymorphisms and idiopathic nephrotic syndrome in Slovak children: preliminary results. Med Sci Monit.

[122] Han SS, Xu YQ, Lu Y, Gu XC, Wang Y (2017). A PRISMA-compliant meta-analysis of MDR1 polymorphisms and idiopathic nephrotic syndrome: susceptibility and steroid responsiveness. Medicine (Baltimore).

